# RANK-ligand inhibition to combat sarcopenia with underlying osteoporosis: a study protocol for a randomized, double-blind, double-dummy, active-controlled trial

**DOI:** 10.1186/s13063-025-08976-7

**Published:** 2025-08-04

**Authors:** Ronald Man Yeung Wong, Pui Yan Wong, Chaoran Liu, Ning Tang, Raymond Chung Wai Wan, Wing Hong Liu, Can Cui, Ning Zhang, Timothy Chi Yui Kwok, Sheung Wai Law, Wing Hoi Cheung

**Affiliations:** 1 Department of Orthopaedics & Traumatology, The Chinese University of Hong Kong, Hong Kong SAR, China; 2 Department of Orthopaedics & Traumatology, Prince of Wales Hospital, Hong Kong SAR, China; 3 Department of Medicine & Therapeutics, The Chinese University of Hong Kong, Hong Kong SAR, China

**Keywords:** RANKL inhibitor, Sarcopenia, Randomized controlled trial, Osteoporosis

## Abstract

**Background:**

Sarcopenia is an age-related disease characterized by gradual loss of muscle strength and muscle mass. Osteosarcopenia is the presence of osteopenia/osteoporosis and sarcopenia, which poses an increased risk of falls and fractures. Currently, there is no Food and Drug Administration (FDA) approved drug for treating sarcopenia. Previous studies showed that nuclear factor-κB ligand (RANKL) inhibition could reduce muscle atrophy and could be a therapeutic target for treating sarcopenia. Denosumab is an anti-osteoporotic drug with RANKL inhibition. This study aims to investigate the effects of denosumab to treat sarcopenia in patients with underlying osteoporosis.

**Methods:**

This study is a randomized, double-blind, double-dummy, active-controlled trial for investigating the efficacy of denosumab in treating sarcopenia in patients with osteosarcopenia. Participants aged 65 years or above with osteosarcopenia will be recruited. Participants will be randomized into the denosumab group or the zoledronic acid group and will be followed up for 1 year. The primary outcomes are muscle strength, muscle mass measured by dual-energy x-ray absorptiometry (DXA) scan, and physical performance. The secondary outcomes are the clinical outcomes, including fall rate, fracture rate and mortality.

**Discussion:**

This study will study the potential therapeutical effects of denosumab (RANKL inhibitor) on osteosarcopenia, which will be crucial given the aging population and expected increase in disease numbers.

**Trial registration:**

ClinicalTrials.gov, NCT06643780. Registered on 16 October 2024. (Retrospectively registered and no protocol changes were made since the recruitment was started for this study).

**Supplementary Information:**

The online version contains supplementary material available at 10.1186/s13063-025-08976-7.

## Background

Osteoporosis is a systemic disease characterized by a decrease in bone mass and disruption of bone microarchitecture. With the aging population, the prevalence of osteoporosis continues to rise. In the United States (US), 10 million individuals over the age of 50 are estimated to have osteoporosis [1]. Unfortunately, the lifetime fracture risk of osteoporotic patients reaches 50% for women and 20% for men. Sarcopenia is the progressive, age-related skeletal muscle disorder that leads to accelerated loss of muscle mass and function [2]. In severe circumstances, individuals can lose up to 50% by the age of 80. The Asian Working Group for Sarcopenia (AWGS) 2019 guidelines is often used for the diagnosis of sarcopenia in Asia, and a clinical study of 1587 participants showed that the general prevalence of sarcopenia in community-dwelling older adults 65 years old or above reached 40% [3]. In patients with fragility fractures, the occurrence of sarcopenia reaches even higher, with up to 95% in males and 64% in females [4]. More importantly, similar to osteoporosis, it is well established that sarcopenia significantly increases the risk of mortality, as there is a high risk of fractures, and a recent meta-analysis showed an increase by approximately fourfold [5]. Both osteoporosis and sarcopenia are strongly associated with falls, fractures, repeated hospital admissions, and mortality [6, 7]. Therefore, both issues in older patients are of huge importance, but are often underestimated as there is no immediate life-threatening issue.

Both muscle and bone loss often coincide, and numerous recent studies have shown that sarcopenia has a bidirectional relationship with osteoporosis. In fact, individuals with sarcopenia have 12.9 times higher risk of osteoporosis as compared to those without [8]. Due to the poor clinical outcomes, the separate entity of ‘osteosarcopenia’ has been coined [8], and is a recognised new geriatric giant in the aging population. Although osteoporosis can be treated with well-established Food and Drug Administration (FDA) approved anti-osteoporotic drugs, despite research efforts, as of now, there is still no FDA approved drug for treating sarcopenia. Furthermore, unfortunately, exercises are poorly tolerated amongst old patients due to adherence and feasibility, causing sarcopenia and disability to persist [9]. Therefore, treating sarcopenia is an important therapeutic step, but also a missing gap [10, 11].

Age-related muscle and bone loss have complex interactions and crosstalk, and several factors have been studied including the receptor activator of nuclear factor-kB ligand (RANKL)/RANK axis. RANK, a tumour necrosis factor receptor superfamily protein in osteoclasts, binds to RANKL causing differentiation of pre-osteoclasts to osteoclasts. RANK is also expressed in skeletal muscle, and interaction of RANKL/RANK causes a pro-inflammatory pathway that inhibits myogenic differentiation and enhances the ubiquitin proteasome system to induce muscle atrophy [12]. Current understanding of muscle-bone interactions is still under research for novel drug therapies. Denosumab, a US FDA approved drug for the treatment of osteoporosis, is a human monoclonal antibody that directs against RANKL [13]. A recently published pre-clinical study that used an osteosarcopenic mouse model, which knocked out the transcription factor of peroxisome proliferator-activated receptor β, leading to low muscle mass and reduced power, fat infiltration into muscle, together with decreased bone mineral density, showed that RANKL inhibition restores muscle dysfunction by increasing muscle volume and force. Gene expressions for myosin I and II were also increased with decrease of anti-myogenic/inflammation marker myostatin [14]. Clinically, there have also been cohort studies that have seen the potential effect of RANKL inhibition in improving muscle parameters [14, 15]. The RANKL/RANK pathway appears to be highly relevant in sarcopenia, and randomized controlled trials are warranted to assess the potential benefit of RANKL inhibitors in the treatment of osteosarcopenia. The objective of this study was to conduct a randomized, double-blind, double-dummy active controlled trial to determine the efficacy of denosumab in treating sarcopenia with underlying osteoporosis.

## Methods

### Study design

**Fig. 1:**
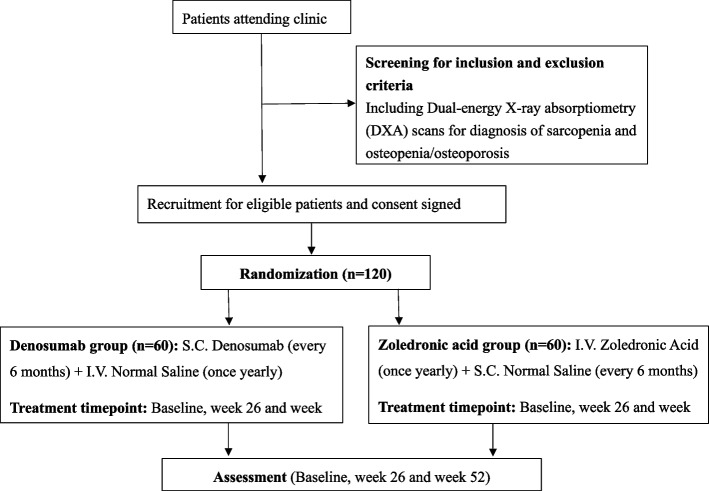
Flow chart of study recruitment. ASM, appendicular skeletal muscle; QMS, quadriceps muscle strength; SF-36, Quality of life Short Form-36; PASE, physical activity scale for elderly; FFQ, food frequency questionnaire

**Fig. 2:**
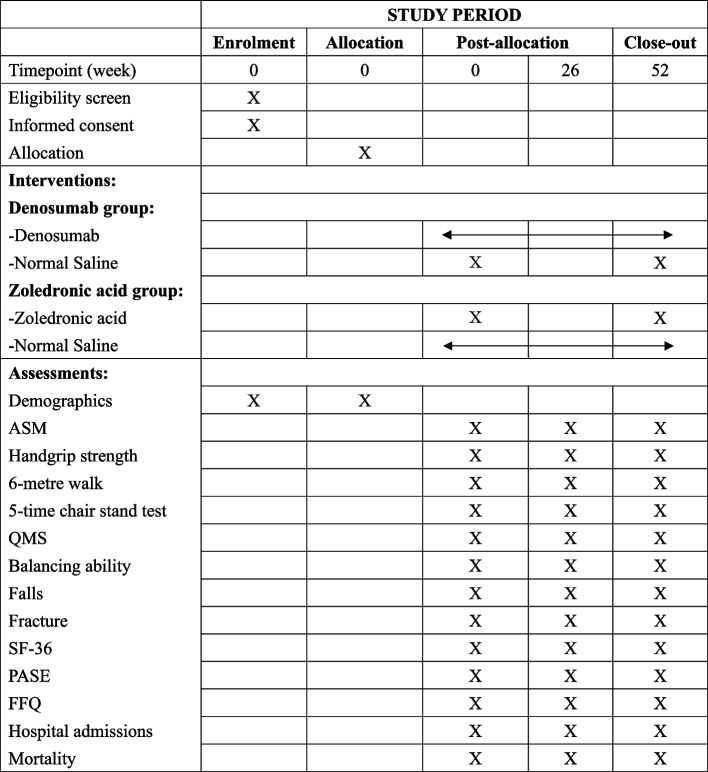
Standard Protocol Items: Recommendations for Interventional Trials (SPIRIT) figure

This is a randomized, double-blind, double-dummy, active-controlled trial to compare the effects of RANKL inhibition with denosumab in treating sarcopenia in osteosarcopenic patients with zoledronic acid as active-control drugs, which are two approved drugs for treating osteoporosis. This is a planned 2-year parallel study in which recruited patients will be randomized and treated by (i) subcutaneous denosumab (RANKL inhibitor) every 6 months (active agent) and intravenous normal saline (NS) placebo yearly (dummy agent) or (ii) intravenous zoledronic acid (active-control) yearly with subcutaneous NS placebo (dummy agent) every 6 months, for 1 year (Fig. 1). The Standard Protocol Items: Recommendations for Interventional Trials (SPIRIT) [16] figure and checklist were provided in Fig. 2 and the supplementary file, respectively. If any amendments to the protocol are to be made, the principal investigator will notify the (i) Joint Chinese University of Hong Kong—New Territories East Cluster Clinical Research Ethics Committee, and (ii) Drug Office, Department of Health, The Government of the Hong Kong Special Administrative Region, and (ii) Health and Medical Research Fund, Health Bureau, The Government of the Hong Kong Special Administrative Region. Any deviations from the protocol will be fully documented using a formal report form. The protocol of the clinical trial registry will also be updated accordingly.

### Study participants

This study will enrol 120 osteosarcopenic patients (*n* = 120) from specialist outpatient clinics or orthopaedic wards from the Prince of Wales Hospital, Hong Kong. Patient enrolment started in April 2024 and is expected to end in April 2026. The coordinating centre includes the Evidence-Based Orthopaedics Clinical Education and Research Program (EBO Program) from the Department of Orthopaedics & Traumatology, The Chinese University of Hong Kong. The composition includes an experienced team with the Director, senior and international advisors, and trained research staff to aid in the coordination and conduct of the clinical trial (https://www.ort.cuhk.edu.hk/research-ebo.html). The trial steering committee is composed of the principal investigator and co-investigators and will meet with the EBO Program monthly for the progress of the trial. Trained research assistants from the team will screen patients according to the inclusion and exclusion criteria. Inclusion criteria are as follows: (1) old males or females aged 65 years or older; (2) diagnosed with osteosarcopenia (sarcopenia diagnosis based on AWGS 2019 guidelines—low appendicular skeletal muscle mass (ASM) by dual-energy x-ray absorptiometry (DXA) (M: < 7.0 kg/m^2^, F: < 5.4 kg/m^2^) AND low handgrip strength (M: < 28 kg, F: < 18 kg) OR low physical performance (6-m walk: < 1.0 m/s or 5-time chair stand test ≥ 12 s); osteopenia/osteoporosis diagnosed based on World Health Organization (WHO) criteria with DXA scan *T*-score ≤ − 1.0) (3); (3) willing and able to comply with study protocol including follow-up evaluations. Exclusion criteria are as follows: (1) history of recent fracture, i.e. within 3 months; (2) history of prior anti-osteoporotic drug; (3) disease or medication affecting bone or muscle metabolism; (4) Chairbound or bedbound; (5) unable to agree to consent; (6) contraindication to drug, i.e. denosumab or zoledronic acid; (7) underlying malignancy or disease known to cause cachexia; (8) severe renal impairment, e.g. creatinine clearance < 35 ml/min; (9) moderate to severe liver failure (Child–Pugh Class B or C). Patients or their representatives are requested to sign written informed consent to participate in the study by the trained research assistants. Monthly meetings will be held for team members to report progress and solve recent problems.

### Public and patient involvement

The initial protocol was designed by the principal and co-investigators with advice from the EBO Program. Further amendments may be made according to performance and feedback from participants, which can potentially improve the study design as well as positivity of participants.

### Sample size calculation

In this study, sarcopenic parameters of muscle strength/function are the primary outcome. A sample size of 48 per group (96 in total with both groups) will have 80% power to detect a significant difference using two-way repeated-measures ANOVA with 0.05 significance level (PASS 11.0, NCSS, LLC, USA). Assuming a drop-out rate of 15%, *n* = 56 per group is required for the randomized controlled trial (RCT) study. By rounding to *n* = 60 per group, a total sample size of *n* = 120 is finalized.

### Randomization and masking

According to our previously established protocol, a computer-generated set of random allocations is sealed in consecutively numbered opaque envelopes by an independent central technician. Once consent is obtained, the patient is randomized (1:1) to (1) 60 mg subcutaneous denosumab every 6 months (active agent) and intravenous normal saline (NS) placebo (dummy agent) once yearly (*n* = 60) or (2) intravenous zoledronic acid (active-control) once yearly and subcutaneous NS placebo every 6 months (*n* = 60). Both denosumab and zoledronic acid are FDA-approved drugs that are recommended for the treatment of osteoporosis [7]. Patients, investigators, and outcome assessors are blinded to treatment assignment. An initial interim report at 2 months will be submitted to Health and Medical Research Fund, Health Bureau, The Government of the Hong Kong Special Administrative Region. The usual interim assessments and reports will be conducted at 1 year. The blinding will only be partially unmasked for the independent data monitoring committee that reviews the interim data for safety purposes. Masking is achieved by ensuring the active drug and corresponding placebo (normal saline) have identical appearance. Intravenous agents for infusion are given with a blanket on top for masking, and subcutaneous agents are given with a covered syringe. These steps are performed to ensure the patient, investigators, and outcome assessors are blinded. The drug is administered by an independent nurse at the day centre. The allocated intervention will be revealed to the participants after completing the 1-year study when they visit the outpatient clinic for a face-to-face follow-up assessment or by phone call. Participants will visit a total of 3 times (baseline, 26 weeks and 52 weeks) during the participation in the study.

### Procedure

As with international guidelines and standards, all osteosarcopenic patients undergo weight-bearing exercises and advise, and receive calcium 1000 mg and 800 IU vitamin D daily. The dosages of calcium and vitamin D are within the recommendations as per local guidelines for the treatment of osteoporosis [17]. Based on randomization, either (1) 60 mg subcutaneous denosumab every 6 months (active agent) and intravenous normal saline (NS) placebo (dummy agent) once yearly (*n* = 60) or (2) intravenous zoledronic acid (active-control) once yearly and subcutaneous NS placebo every 6 months (*n* = 60) will be administered. Compliance to the procedure is documented for each patient by an independent personnel. Patients will be asked about adverse events and concomitant medications on each follow-up by the blinded clinician. Patients will receive reminder messages on the phone or phone calls before each follow-up visit. Each patient will receive HKD100 supermarket coupons (~ US12.8) after completion of the baseline assessment. Upon complete assessment for the remaining time points, another HKD150 (~ US19.2) supermarket coupons will be received. These are the incentives for the participants to be enrolled in the study. After the trial, the patients will be unblinded to the intervention and told the results of their assessments measured within the study period by the investigators. Any subsequent modifications of study procedures will be reported to the relevant committees as stated above for approval.

### Primary outcomes

(A) Six-metre walk (baseline, 26, and 52 weeks): time taken to walk 6 m without deceleration [3]. Average result of 2 trials is recorded. Slow speed is defined as < 1.0 m/s.
(B) Five-time chair stand test (baseline, 26, and 52 weeks): the time to rise from a chair 5 times is recorded [18]. The cut-off is taken at ≥ 12 s.
(C) Appendicular skeletal muscle mass (ASM) (baseline, 26, and 52 weeks): determined with dual-energy x-ray absorptiometry (Horizon®, DXA system, Hologic, USA) [3]. Total ASM by DXA is evaluated by segmented measurement of muscle mass at four limbs by operator-defined cutlines at specific anatomical landmarks. ASM is adjusted to the square of height to calculate Appendicular skeletal muscle mass index (ASMI) (kg/m^2^). Low ASMI by DXA is < 7 kg/m^2^ for men and 5.4 kg/m^2^ for women [3].
(D) Handgrip strength (baseline, 26, and 52 weeks): assessed by spring-type hand dynamometer (JAMAR Hand Dynamometer 5030JO) [3]. Cut-off for men is < 28 kg, and for women is < 18 kg. Maximum reading of 3 trials using the dominant hand in a maximum-effort isometric contraction is taken.
(E) Quadriceps muscle strength [baseline, 26-, and 52-weeks]: measured on affected limb with isometric dynamometer (Baseline, Genova, Italy). Subject will sit on a chair with both feet above ground, while raising the affected leg 45° forwards. The dynamometer is placed above the ankle and the subject will push the leg forward with maximum force. Measurements will be repeated 3 times and maximum value will be used for evaluation [19].
(F) Balancing ability (baseline, 26, and 52 weeks): The Basic Balance Master System (NeuroCom International Inc, USA) is used to measure static and dynamic ability of subjects to maintain center of balance. Subjects will stand barefoot on the force plate and control the location of their center of gravity by weight-shifting to eight different targets. Measured parameters of limits of stability test include reaction time(s), directional control (%), movement velocity (degrees/s), endpoint excursion (%), and maximum excursion (%). The system is a reliable tool for objective assessment of balancing ability for older patients [19].

### Secondary outcomes

(A) Falls (1 year): To assess the occurrence of falls, patients are required to self-report via a fall calendar, which will be returned at 1 year. Calendar reporting has been well proven to be reliable for fall studies [19].
(B) Fracture (baseline, 26, and 52 weeks): Assess occurrence of a fracture. Patients are required to self-report and checked from the Clinical Management System (CMS).
(C) Quality of life Short Form-36 (SF-36) (baseline, 26, and 52 weeks): is a widely accepted, well-validated functional status questionnaire to measure health-related quality of life in eight domains, including physical functioning, health perception, etc. [19].
(D) Hospital admissions (1 month, 3 months, 1 year): number and cause of emergency hospital admission are documented [20].
(E) Mortality (1 month, 3 months, 1 year): (common clinical time-points for clinical studies): Mortality is documented from the CMS.
(F) Bone mineral density (BMD) values (baseline, 26, and 52 weeks): BMD values and T-score of lumbar spine and hip are measured by DXA.

### Confounding variables

(A) Physical activity scale for elderly (PASE) (baseline, 26, and 52 weeks): is well-validated for use in older populations [21]. PASE will be used to assess the physical activity level.
(B) Food frequency questionnaire (baseline, 26, and 52 weeks): daily and weekly intake of 280 food items will be performed using a validated food frequency questionnaire developed in a local population survey [11]. Mean nutrient quantitation and energy intake per day will be calculated referring to food composition tables derived from the Chinese Medical Sciences Institute and Centre for Food Safety in Hong Kong.

### Safety monitoring

The patients will be monitored continuously in each follow-up assessment. In case the patients report harms or adverse events related to the assigned drug groups or request a change to the other drug, they will be switched to another drug group only after the clinicians check their conditions and consider they are suitable for switching the group. The principal investigator or the ethics committee may terminate the clinical trial if necessary due to any safety issues or concerns. The serious adverse events (SAE) reported by the patients or observed by the investigators will be documented and will be reported in a separate form to the independent data monitoring committee. If the patients are not willing to participate in this project, they can refuse or withdraw from it, which will not impact the quality of health care. In the event of patient withdrawal, they will be unblinded to the intervention and the clinicians will reveal their assigned group at the follow-up visit. The EBO program involved personnel and trial steering group will meet to review the trial conduct monthly. The Drug Office, Department of Health, The Government of the Hong Kong Special Administrative Region, and Health and Medical Research Fund (HMRF), the Health Bureau, The Government of the Hong Kong Special Administrative Region, and the Ethics Committee will receive a yearly progress report.

### Data processing and analysis

All study documents will be stored and locked in a room, and the data will be kept in an electronic database with limited access during the study period and after the study. Only the principal investigator and research coordinators will have access rights to the study documents and the electronic database. The electronic database will also be encrypted. Double data entry will be used to ensure the quality of data. All quantitative data will be expressed as mean ± standard deviation. Statistical analysis will be performed using SPSS (IBM, NY, USA). Data analysis will be performed based on the intention-to-treat principle, including all randomized participants. One-way (for between-group differences in over-time changes) and group repeated measures ANOVA with post-hoc Bonferroni analysis, as well as independent/paired *t*-tests, will be performed. Nonparametric tests are used when the data is not normally distributed. The general linear model will be used to explore the follow-up effects of treatments by adjusting for baseline, recent nutrition, and exercise status. The significance level is set at *p* < 0.05 (2-tailed). For missing data, the last observation carried forward method will be used for replacing the missing responses. The baseline characteristics of those being followed up and those who are loss to follow up will be compared for any statistical difference.

## Discussion

The annual costs of osteoporosis were estimated to be 17 billion USD in the US in 2005, and 37 billion Euros in Europe in 2010 [1]. The major consequence of osteoporosis is fractures that often result from low-energy trauma, which leads to heavy costs and high risk of morbidity and mortality [22]. As for sarcopenia, the disease attributes to a huge socioeconomic burden and costs reach more than 18.5 billion USD in the US yearly [5]. As the occurrence of both osteoporosis and sarcopenia concomitantly is common, this study is designed as a pragmatic issue clinically.

The mainstay of treatment of sarcopenia is exercises and nutrition, but pharmacological agents would be useful for patients that do not respond or are unable to adhere [23]. There has been increased research efforts in identifying new therapies; for example, steroid hormones, but the use has been limited due to adverse side effects including risk of cardiovascular events [24]. Inhibition of RANK ligand is a novel concept in treating sarcopenia. A clinical study comparing the effects of denosumab with other anti-resorptive agents identified an association that denosumab demonstrated significant improvements in muscle parameters after 5 years of completion and had significant reduction in fall rates [25]. However, this was not a randomized controlled trial, and there are very limited studies especially in Asians. This study will investigate the effect of RANKL inhibition on sarcopenia in terms of physical performance, muscle mass, and muscle strength and also follow up on clinical outcomes including fall rates and mortality as a randomized controlled trial.

The strength of this study includes performing a randomized double-blind, double-dummy, active-controlled trial for study design which can reduce biases and study the effects of RANKL inhibition on sarcopenia more accurately compared with cohort studies. There are also multiple time points for assessments, which can monitor the change of muscle parameters and clinical outcomes during the 1-year study period. The DXA, which is the gold standard for the assessment of body composition, will be used to compare the effects of the pre- and post-treatment, which could provide accurate and reliable results. There are several anticipated limitations in our study; as this study requires assessment at several time points, there may be loss to follow-up or drop-out. To increase the follow-up rate, incentives in terms of supermarket coupons will be provided at the baseline when they are willing to participate in the study and at the final follow-up assessment if they finish all follow-up time point assessments. It is used to engage the participation and has no link with nutritional support.

In summary, this study will study the potential therapeutical effects of denosumab (RANKL inhibitior) on patients with sarcopenia and underlying osteoporosis, which will be crucial given the aging population and expected increase in disease numbers. The effects of denosumab on sarcopenia can have great potential in reducing the costs caused by osteosarcopenia and improving the quality of life of these people.

## Trial status

Protocol version 1 (9 April, 2024). Recruitment of participants for this study started on 9 April 2024, and is expected to end on 9 April 2027. The recruitment is ongoing.

## Supplementary Information

Supplementary Material 1.

## Data Availability

Not applicable.

## Abbreviations

FDA: Food and Drug Administration
RANKL: Nuclear factor-κB ligand
DXA: Dual-energy x-ray absorptiometry
US: United States
AWGS: The Asian Working Group for Sarcopenia
NS: Normal saline
RCT: Randomized controlled trial
ASM: Appendicular skeletal muscle mass
CMS: Clinical Management System
SF-36: Quality of life Short Form-36
PASE: Physical activity scale for elderly
